# Character of Amalgams

**Published:** 1874-03

**Authors:** 


*Character of Amalgams.*—Dr. E. A. Bogue, in a paper read before the New York Odontological Society, gives the following information concerning the character of the amalgams in common use:

" The copper amalgam, which is quite difficult to prepare, is dark in color, and its oxide is poisonous. Though extensively used in Germany, it is fortunately seldom or never employed in this country.

The palladium amalgam is quite as black as copper, and cannot be used except in the form of a precipitate; but it is not liable to more than superficial oxidation, and that entirely innoxious.

While most metals combined with mercury are supposed to constitute merely mechanical admixtures or solutions, palladium forms a true chemical union attended by the evolution of heat.

This amalgam is very highly esteemed in certain quarters in England; but its costliness, blackness and brittleness, however, prevent its being generally adopted.

The amalgams which have been most in favor in this country, have been, until within a few years past, composed principally of silver and tin.

Among these, however, are some that exhibit traces of other metals; such as Townsend's and Lawrence's, and perhaps a few others, which contain the copper that enters into the composition of the coin silver they use, and Holmes', which contains a small proportion of gold that is added to diminish the shrinkage.

Recently, through the researches of Messrs. Fletcher and Tomes, a new amalgam alloy has been introduced, which is composed of gold, platinum, silver and tin. The office of the silver is to harden; of the gold, to lessen contraction and oxidation; of the platinum, to hasten the setting and preserve the color. All these are essential points, and more essential in England, perhaps, than elsewhere, because an amalgam filling there, as a rule, receives its entire finish at the time of its insertion.

This filling hardens more rapidly than any other which has been in common use; and its contraction is less than that of any other *compound* filling whose contractions have been accurately measured.

As to the shrinkage: It has been thought by some, that the action of certain amalgams in the tooth, drawing together and rising toward the centre, was indicative of expansion. But it may be accounted for by the tendency of some metals to assume the form of a spheroid; and the amalgams which harden most slowly are especially inclined to this shape.

Besides, the experiments which havs been made upon amalgams by the specific gravity test, have exhibited marked shrinkage.

According to a paper read by Mr. Tomes, before the Odontological Society of Great Britain, March 4th, 1872, the variation is from .037 of a unit in the case of palladium to .38 of a unit in the case of tin and silver used in equal parts; the shrinkage amounting to ten times more in the latter case than in the former.

The shrinkage of copper also is very little—scarcely greater than that of palladium.

It has been claimed, however, that the specific gravity test is not sufficiently accurate to measure the shrinkage of these substances ; but a mechanical apparatus, constructed for the purpose of testing the very minute expansion and contraction of bodies, furnishes substantially the same results."

